# Why Death Is Most in One's Self‐Interest, and Necessarily So

**DOI:** 10.1111/bioe.70059

**Published:** 2025-12-26

**Authors:** Victor Kriska

**Affiliations:** ^1^ Department of Philosophy Bowling Green State University Bowling Green Ohio USA

**Keywords:** death, deprivationism, dying, Epicureanism, omega elixir, thanatism

## Abstract

Most of us think that death is usually not in the self‐interest of the one who dies. Let us momentarily put this belief aside and examine death in a new light. This paper presents a two‐step argument to show why death is most in one's self‐interest, necessarily. The first step contends that death minimizes one's bad at no loss of one's good, such that if one's life has or will have any bad whatsoever, death is most in one's self‐interest. The second step maintains that all possible self‐interested lives have extrinsic bad given their potential to be better. Hence, death is most in one's self‐interest, necessarily. This paper challenges prevailing notions on death and remains fruitful even for those readers disinclined to accept its conclusion. Attempting to determine where the argument goes awry provides an opportunity to sharpen one's own views on death.

If, on the one hand, I were offered the fortune and fame of Cæsar or Alexander, free from the least stain; and, on the other hand, death to‐day, I should unhesitatingly choose to die to‐day.

—Giacomo Leopardi, Essays and Dialogues

## 1 Introduction

Consider:

*Blissful Life*. Suppose there exists a being, Y, who enjoys a life of astonishing good. This life is, and forever will be, entirely pleasant and satisfying with just one exception: Y is destined to soon feel the pain of a brief pinprick. In advance, Y is presented with the “omega elixir”, a concoction that quickly and painlessly results in death for whoever drinks it. Y is fully self‐interested and must now make the choice to either do nothing and endure the pinprick or drink the elixir and thereby avert the impending pain. Assume Y completely understands the magnitude of the situation and that deliberation over this decision does not have a bad effect on Y.^1^

Most will hold that it is not in the self‐interest of Y to drink the omega elixir. The pain of the pinprick is such a minor bad, the thinking goes, that it is immeasurably compensated for by the good in Y's life. Many would generalize that eliminating an abundance of one's good to avoid a minor bad is repugnant to self‐interest. Thus, as Y is fully self‐interested, Y must do nothing and endure the pinprick.

This majority view is mistaken. Given that the action of the elixir is both quick and painless, Y is compelled by self‐interest to drink it. In explaining why, I will argue for the view that death is most in one's self‐interest, necessarily. Let us call this view “hard thanatism”.^2^

Hard thanatism has two defining features. First, it holds that whenever death is in one's self‐interest, it is *most* in one's self‐interest. Second, it holds that death is in one's self‐interest *necessarily*, which is to say, in every possible case. Together, these features entail that it is impossible for there to exist a self‐interested life where death is not prudentially best for the one who lives it, regardless of the quality and content of that life.^3^ This makes hard thanatism the strongest form of thanatism—the view that death is in one's self‐interest—to ever be advanced.^4^

The task of this paper is to make the initial case for hard thanatism.^5^ I recognize that on the face of it this view seems absurd, but I will show how its animating force flows naturally from a handful of principles that many will be tempted to accept. The paper proceeds as follows. Section 2 clarifies some commitments of hard thanatism. Section 3 examines principles that undergird the hard thanatism argument. Section 4 presents the argument. Section 5 concludes. The remainder of the paper does not examine other (softer) forms of thanatism. For ease of reference, every following appearance of “thanatism” is shorthand for “hard thanatism”.

## 2 Clarifications

Since thanatism is admittedly an unusual position, it is appropriate to clarify, in brief, some of its commitments.

### 2.1 Self‐Interested Beings

Thanatism is restricted to beings who have a self‐interest. Death cannot be most in the self‐interest of beings lacking a self‐interest, because, of course, nothing can be of self‐interest for those beings. Thanatism pertains to all sentient life, as it is generally in the self‐interest of sentient beings to avoid bad and seek good. Non‐sentient beings such as bacteria plausibly have no self‐interest. If that is right, then those beings are excluded from the scope of this position.

Thanatism denies that a being can be self‐interested in any way after death. Instead, the position makes a claim regarding the possible ways a being's life can go. The claim is that obtaining death at time t1 is a prudentially better option for one than all of one's possible lives where one obtains death after t1. The relevant comparison is therefore not between life and death, but between a shorter life and the very same shorter life (without death) plus more life.

I take it this is what people typically mean when they delicately express the sentiment that a terminally ill person in excruciating pain would be better off dead. It is not to suggest the person somehow exists while dead to benefit from death, but that in a comparison between the person's life ending and it continuing, the former option better serves the self‐interest of the person while alive. Thanatism expands the notion of “better off dead” to include any possible self‐interested being, not solely those finding themselves in exceedingly dire circumstances. It asserts that in every case, the shorter life is prudentially better than the longer one.

### 2.2 Death Versus Dying

Thanatism concerns death, not dying. To illustrate why this distinction is significant, consider:

*Reward Rob*. Rob is presented with three buttons. He is correctly told that pressing Button A will reward him with a massive fortune, pressing Button B will reward him with a paltry sum, and pressing Button C will “reward” him with nothing at all. He is also informed of a twist: pressing Button A will give him an excruciating and sustained electric shock (after which the pain will end, and the reward will be given), while pressing the others will not. Rob is obliged to press one and only one button. He is fully self‐interested and must now decide what to do.

Button A holds what is prudentially best for Rob.^6^ Still, if the shock is too severe, then it is prudentially advisable to settle for the paltry sum rendered by Button B. This does not mean Button B contains the superior reward. It is far inferior. It simply means that the difficulty of obtaining what is prudentially best pushes Rob to settle.

Putting this scenario in the context of thanatism, death corresponds to the reward behind Button A, while dying, in some cases, is like the electric shock. If a particular manner of dying is sufficiently unpleasant, it is prudentially advisable to avoid it. This observation has no bearing on how death itself (that is, a shorter life) compares against all other (longer life) alternatives. For thanatism, death continues to be prudentially best even when it is unapproachable due to ugly practical realities.

I refrain from developing an account of when it is prudentially advisable to undergo the dying process in order to focus on establishing the thanatist position. Merely note that if thanatism is true, current views toward dying need to be recalibrated.

### 2.3 Self‐Termination

Thanatism alone is not an argument for self‐termination.^7^ It may seem that if death is most in one's self‐interest, then self‐termination must be advisable. This leap is erroneous for at least three reasons.

The first reason is self‐regarding and relates to the death versus dying distinction. Any real prospect to self‐terminate cannot isolate death from what must be carried out to reach it. An attempt may produce considerable bad for one, from negative consequences in sourcing an appropriate method to pain endured in the dying process. Further, many methods carry a substantial degree of risk. They can be unreliable such that the combined likelihood and severity of failure prudentially precludes one from utilizing them. Nonetheless, if one has a reliable, quick, and painless method at hand, such as our imagined omega elixir, then one is compelled by self‐interest to complete self‐termination.

The second and third reasons are other‐regarding. Unless we are egoists, the fact that something is compelled by self‐interest (as going through the dying process sometimes is) does not by itself imply we should act on it. The second reason acknowledges that there plausibly exists a pro tanto duty to not abandon one's relations. If, for example, one receives an offer for a dream job that requires relocation far away from one's care‐dependent parents, then one has a defeasible yet strong reason to reject the offer. Similar is the case with self‐termination. The second reason is especially salient with regard to one's close relations, but also appreciates the unfortunate impacts self‐termination has on members of the broader community. The third reason envisions what good one can do in the world. There are morally important projects that demand individual contributions to progress. Self‐termination excludes one from contributing or potentially contributing to morally worthy, other‐regarding projects.

For these three reasons, and perhaps others, self‐termination is generally inadvisable even when thanatism is accepted.^8^

## 3 Principles

The animating force of the argument for thanatism rests on three respective principles concerning self‐interest, death as annihilation, and temporal existence. I will present and motivate each principle, but will not argue for them decisively. If one denies one or more of the principles, then the argument will be unconvincing. However, the principles have, I believe, broad intuitive appeal. Even if one does not accept them all, one can at least feel their pull and see where they lead.

### 3.1 Self‐Interest Principle

The Self‐Interest Principle (SIP) maintains:

SIP: (1) What is most in one's self‐interest is what is prudentially best for oneself. (2) If an option (A) minimizes one's bad (if there is any at all) and results in no loss of one's good or (B) maximizes one's good and results in no incurrence of one's bad, then (C) that option is prudentially best for oneself.

(1) is definitional. If an option is most in one's self‐interest, then it is prudentially best for oneself, and if it is prudentially best for oneself, then it is most in one's self‐interest. In (2), (A) and (B) each set a sufficient condition for occasions when an option is prudentially best for oneself. These conditions, I take it, are definitionally true. If, per (A), an option *truly* minimizes one's bad and results in no loss of one's (current or future) good, then it is most in one's self‐interest. This condition will rarely, if ever, be met in ordinary cases. Virtually everything has an opportunity cost. If one attempts to minimize one's bad (of boredom and so forth) by spending a pleasant evening reading, one loses out on the good of a delightful stroll in the park. This is a loss of good, and so (A) is not satisfied. It is likewise the case with (B). If one attempts to maximize one's good by having enjoyable conversation with friends, one incurs the extrinsic bad of not engaging in some other pleasing activity. This is an incurrence of bad, and so (B) is not satisfied.

### 3.2 Annihilation Principle

The Annihilation Principle (AP) maintains:

AP: Death is the annihilation of the one who dies.

For this paper, let us constrain this principle to self‐interested beings. AP has credible empirical support and is widely accepted in the philosophy of death literature.^9^ There appears to be a strong correlation between one's body and mental state. A temporary arrest of certain bodily functions, such as when one is placed under anesthesia, results in a temporary cessation of consciousness. A permanent arrest of those same functions, as coincides with death, plausibly results in a permanent cessation of consciousness. This reduces to the annihilation of the self‐interested being who dies.

### 3.3 Existence Principle

The Existence Principle (EP) maintains:

EP: For something to be good or bad, better or worse, for a self‐interested subject S at time t, S must exist at t.

In other words, if predicates such as “is good” or “is better” are to be properly ascribed to a self‐interested subject at a particular time, then that subject must exist at that time.^10^ The plausibility of EP is well illustrated by example.

Suppose one receives a pinprick at noon, and it hurts for exactly five minutes. The pain of the pinprick is bad for one during those five minutes, and one is made worse off by the pain during that time. This is the right result and is accounted for by EP. In the time one is worse off for experiencing this bad, one exists.^11^

Now suppose a different case. Toxic waste is illegally dumped near a village ten years before a child, Alice, is born. Shortly after her birth, her family moves into the village, and while a child, she is stricken with a debilitating illness because of her exposure to the toxins. Clearly, the illegal dumping is bad for Alice and her life is worse because of it. But it is not bad or worse for her ten years *before she existed* when the waste was originally dumped. At that time, we did not have an “Alice” subject to whom badness or worseness could be ascribed. It is bad and worse for her when she actually exists and not ten years before her birth when there is no Alice at all. The situation is likewise after Alice dies. The toxic waste is not bad or worse for her ten years *after she existed* even though it *was* bad and worse for her while she was alive. This is a sensible picture and what we would expect if EP were true.

One might worry that EP is in tension with the widespread belief that it can be better for a being not to have been. After all, per EP, it is unintelligible to profess “better” for a being that is never created. We can reconcile this apparent tension by reflecting on what “better not to have been” expresses. It should not be taken as the claim that had a being never been created, there would exist a literal entity whose nonexistence is enriched. Rather, I propose the heart of this idea, as usually voiced, is the tacit recognition that death is most in a being's self‐interest, and so it is in some crucial way regrettable that the being came into existence. Interpreted through this lens, the apparent tension evaporates.^12^

Elements of EP trace back to Epicurus. He argued that death is inconsequential for the one who dies because one does not experience death while alive, and once dead, there no longer exists a subject for whom death (or anything else) can be good or bad.^13^ The most common objection to the Epicurean argument comes from deprivationism. Deprivationism holds that death is bad for one insofar as it deprives one of potential good life.^14^ It identifies the putative badness of death as extrinsic, that is, what one loses out on as a result of one's death. It accepts that death has no intrinsic good or bad; this is what motivates the appeal to extrinsic good and bad in the first place.

Defenders of deprivationism deny EP, but we should be hesitant to acquiesce. If we accept from deprivationism that there is no intrinsic good or bad for the deceased, then it is a small step to accept that there is no extrinsic good or bad for the deceased as well. In both cases, there no longer exists a subject for whom things can be good or bad, intrinsically or extrinsically. Just as there is no subject to enjoy intrinsic good, there is no subject to lose out on good.

## 4 Argument

With these three principles in hand, we are equipped to lay out the argument for thanatism. It is as follows. Once one dies, one no longer exists (AP), and so nothing can be good or bad or better or worse for one from that point onward (EP). Death thereby minimizes one's potential bad *and good* to the greatest extent possible by completely preventing both. So far, the argument is relatively uncontroversial, though it is unclear how thanatism will be reached. Death minimizes one's bad, but also needs to result in no loss of one's good to be most in one's self‐interest (SIP). Yet the prevention of one's good appears to be a loss.

I contend that the prevention of one's good by means of death can never lead to a loss for oneself. The concept of loss presupposes that things can be better or worse. This applies to both intangible and tangible things. Consider, for example, how one can lose a valued memory, friend, or monetary sum, but cannot lose a house fire, tuberculosis, or unwanted pocket lint. Accordingly, at least two requirements must be met for something to genuinely be a loss for oneself: (1) that one does not or will not have the thing, and (2) it would be better if one did, other things equal. This second requirement goes unsatisfied when one's good is prevented by death. At the precise moment of the prevention, nothing can be better for oneself (AP and EP). Death prevents one from enjoying one's good, but it never causes one to lose or lose out on it.^15^ It makes no difference how much good one's life contains or potentially will contain when thinking about the prudential nature of death because good is never lost for the one who dies.^16^ Death minimizes one's bad and results in no loss of one's good. Thus, if one has any bad at all, death is most in one's self‐interest (SIP). Put more formally:

(P1₁) What is most in one's self‐interest is what is prudentially best for oneself. If an option minimizes one's bad (if there is any at all) and results in no loss of one's good or maximizes one's good and results in no incurrence of one's bad, then that option is prudentially best for oneself.

(SIP)

(P2₁) Death is the annihilation of the one who dies.

(AP)

(P3₁) For something to be good or bad, better or worse, for a self‐interested subject S at time t, S must exist at t.

(EP)

(P4₁) Nothing can be good or bad, better or worse, for one once one dies.

(Follows from 2 and 3)

(P5₁) One's death minimizes one's potential bad to the greatest extent possible.

(Follows from 4)

(P6₁) One's death also minimizes one's potential good to the greatest extent possible.

(Follows from 4)

(P7₁) However, there is no ensuing *loss* of one's good from this minimization. If there was a loss of good, that would entail it is better for the one who dies to have it. Nothing can be better for one once death is obtained. Death prevents one's good only at those times in which it is not better to have it.

(Follows from 4 and 6)

(C₁) Death minimizes one's bad without any loss of one's good. Death is most in one's self‐interest (if one has any bad at all).

(Follows from 1, 5, and 7)

This argument is the animating force behind thanatism. It supposes that there is or will be some bad in one's life for death to be prudent. Otherwise, there is no bad to minimize, and no impetus for prudentially favoring death. As an empirical matter, all self‐interested beings in the actual world suffer intrinsic bad. Still, this is insufficient to show that death is *necessarily* most in one's self‐interest. Establishing the full thanatist position requires arguing that all possible self‐interested lives unavoidably possess bad. Let us turn to that argument.

Recall *Blissful Life*. Y faces an intrinsic bad with the pinprick. This bad, as I have contended, is sufficient to make it such that death is prudentially best for Y. Suppose we modify the thought experiment so the stipulation of the pinprick is removed. Y's life remains imbued with good and now neither contains nor will contain any intrinsic bad. It is a *Truly Blissful Life*. Here, intrinsic bad is minimized to the greatest extent, but *extrinsic* bad is not. This is because Y's life could be better.

In principle, the life of any self‐interested being can be improved even if that life abounds with good and forever avoids all intrinsic bad. This could be achieved either by holding the subject static and changing the external conditions to slightly increase the subject's good, by holding the external conditions static and changing the subject to slightly increase the subject's good, or by changing both to slightly increase the subject's good. These slight increases can accumulate ad infinitum to perpetually enhance the amount of good in any self‐interested life. All possible self‐interested lives have the potential to be better. Therefore, all possess extrinsic bad. And through death, this bad is minimized at no loss of good. We have come to the full position of thanatism. Put more formally:

(P1₂) The life of any possible self‐interested being can be made better by either increasing some good‐making feature within that life, increasing the subject's capacity to enjoy good, or both.

(P2₂) If the life of a self‐interested being can be made better, then that life contains extrinsic bad.

(P3₂) The life of any possible self‐interested being contains extrinsic bad.

(Follows from 1 and 2)

(P4₂) If the life of a self‐interested being contains bad (intrinsic or extrinsic), then death is prudentially best for that life.

(C₂) For the life of any possible self‐interested being, death is prudentially best. Death is most in one's self‐interest, necessarily.

(Follows from 3 and 4)

One may object that there is a theoretical upper bound to how much good a life can accrue. Once this upper bound is reached, it would be impossible to make the life any better than it is, and so there would be no extrinsic (or intrinsic) bad to minimize. I am skeptical of the possibility of a non‐arbitrary limit on how much good is possible for life.^17^ Nevertheless, the success of this objection would make little difference. Even a life of perfect good, free of all extrinsic and intrinsic bad, stands equivalent to death with respect to self‐interest. Such a hypothesized life would offer no advantage over death. If it were an advantage, then it would be better for the deceased to have it. Per AP and EP, nothing is or can be better for the deceased. If a life of perfect good were possible, one would have no reason to prefer it over death, just as one would have no reason to prefer death over a life of perfect good.

## 5 Conclusion

The argument for thanatism has been presented. If it succeeds, then death is most in one's self‐interest, necessarily. Many are inclined to resist this conclusion. The path ahead for the opponent of thanatism is clear. One desiring to undermine the animating force of the position must reject one or more of the three principles, or show the first standard‐form argument to be invalid. In the meantime, the responsibility rests on those persuaded by thanatism to develop an account of when it is prudent to undergo the dying process, and to explore other consequences thanatism has for our theorizing and our lives.^18^

## Conflicts of Interest

The author declares no conflicts of interest.

## Endnotes

1 For a related case, see Benatar [1, pp. 48–49].

2 “Thanatism” comes from the Greek θάνᾰτος, meaning death. The following argument for hard thanatism is a companion to my argument for extinctionism. While the arguments are similar, they are distinct in terms of scope and strategy, and each stands on its own merits.

3 As used in this paper, the word “prudent” and its derivatives refer to self‐interest. So, if an option is “prudentially best”, then it is best insofar as self‐interest is concerned.

4 I introduce the term “thanatism”, but note that a variety of existing positions fall under this definition. They range from affirming death as being in one's self‐interest (to varying degrees) only in extreme cases, such as to disburden one of a painful and irreversible disease, to death being so for all or nearly all actual lives. Examples of thanatist arguments are found in Brandt [2], Simmons [3], and Sullivan‐Bissett [4].

5 David Benatar's arguments in support of antinatalism (the view opposing procreation on moral grounds) have been adapted by others to argue for thanatist conclusions, though Benatar rightly insists that antinatalism does not entail any particular view on death [5, pp. 157–160]. I seek to make a case for hard thanatism that does not rely on Benatar's arguments. Even if such a case could be made, Benatar's arguments face formidable challenges from, for example, Harman [6], Weinberg [7], and Magnusson [8]. Note, however, that one can readily arrive at antinatalism *from* hard thanatism. Plausibly, we have a pro tanto duty to oppose the creation of a being when, if created, death is most in that being's self‐interest, necessarily.

6 More precisely, the reward behind Button A is prudentially best for Rob given the three options. What is *really* prudentially best for Rob, as I will argue, is death.

7 I prefer the term “self‐termination” to “suicide” because of the latter's negative connotation.

8 Comparable factors explain the inadvisability of the termination of others. We live within a network of associations. Even if another's termination could somehow be made sufficiently quick and painless, the death of that individual will almost always have serious negative externalities.

9 For a collection of arguments against the existence of an afterlife, see Martin & Augustine [9]. Multiple works assume what is effectively AP. Examples include Bradley [10], DeGrazia [11], Boonin [12], and Häyry & Sukenick [13].

10 EP is similar to, though broader than, Jeff McMahan's Existence Requirement: “A person can be the subject of some misfortune only if he exists at the time the misfortune occurs” [14, p. 33]. McMahan rejects the Existence Requirement because it can lead to the conclusion that death is not bad for the one who dies [Ibid., p. 39].

11 For a related case, see Timmerman [15, p. 407]. After the pain subsides, one could be worse off from the bad memory of the situation or better off by integrating the memory in such a way that culminates in a positive outcome. Still, these repercussions can be actualized strictly when one exists.

12 I offer one avenue to make sense of “better not to have been”, but there are alternatives. Following Joel Feinberg, Benatar writes “[w]hat we mean when we say that somebody would have been better off not having come into existence is that nonexistence would have been preferable” [16, p. 176]. Benatar would presumably challenge my proposed interpretation on the basis that it confuses “a life worth continuing [for the sake of that life]” with “a life worth bringing about [for the sake of that life]” [Ibid.]. If thanatism is right, no relevant distinction exists between the two. The same reason—that death is most in one's self‐interest, necessarily—explains why a life is worth neither continuing nor bringing about.

13 Epicurus puts forth this argument in a concise letter. See Epicurus [17].

14 For an influential defense of deprivationism, see Nagel [18].

15 Of course, parallel reasoning affirms that the prevention of one's bad via death will not result in a benefit. This point is a curiosity and does not, I believe, affect the force of the argument. While prevention of bad in this way does not result in a benefit, one's death also removes the value of having benefit (AP and EP).

16 Note that thanatism is compatible with any plausible axiology. It does not assume the axiology of negative utilitarianism, which assigns intrinsic value exclusively to bad. (For more on negative utilitarianism, see Smart [19] and Knutsson [20].) The argument stands even when good is weighted more heavily than bad. The measure of good is not a factor because good is not lost. Conversely, it may seem that the measure of bad is not a factor because prevention of bad is not a benefit. To reiterate, this becomes true only upon death. Once death is obtained, the question of benefit is rendered null.

17 Ruth Chang argues that one “can have too much of a good thing” [21, p. 11]. If she is right, perhaps it is possible to have an excess of good, though our imagined circumstance of a *Truly Blissful Life* is far afield from the more practical cases she considers. For a recent response to Chang, see Hedden & Muñoz [22].

18 I am deeply grateful to Spencer Case, Wes Siscoe, Michael Weber, and three anonymous reviewers for helpful feedback on earlier versions of this paper.
